# Supplementary material for: Effect of Dietary Difructose Anhydride III Supplementation on the Metabolic Profile of Japanese Black Breeding Herds with Low-Level Chronic Exposure to Zearalenone in the Dietary Feed
Source: Toxins (Basel). 2025 Aug 14;17(8):409. doi: 10.3390/toxins17080409 (PMC12390375; doi:10.3390/toxins17080409)
Supplement: Supplementary file 1 [file toxins-17-00409-s001.zip › toxins-3745173-supplementary.pdf]

Supplementary Materials: Effect of Dietary Difructose Anhydride III Supplementation on the Metabolic Profile of Japanese Black Breeding Herds with Low-Level Chronic Exposure to Zearalenone in the Dietary Feed

Table S1. Results of urinary ZEN concentration and serum biochemical analysis from 3 JB breeding herds as a preliminary monitoring.

**Parameters**	**Herd 1 (*n*=11)**	**Herd 2 (*n*=6)**	**Herd 3 (*n*=3)**	***P*-value**	**Reference value***
ZEN (pg/mg/Cre)	749.3 ± 19.9 (*n*=2)	1885.2 ± 578.6 (*n*=2)	4575.3 ± 927.2 (*n*=2)		
AMH (pg/mL)	952.2 ± 185.1	806.2 ± 107.7	627.0 ± 110.8	0.584	15–2000
SAA (mg/L)	2.5 ± 0.7	1.6 ± 0.4	1.2 ± 0.3	0.471	<6.5
GOT (IU/L)	66.1 ± 2.4	57.2 ± 3.5	57.8 ± 2.7	0.078	35–80
GGT (IU/L)	15.6 ± 1.0	12.8 ± 1.2	15.5 ± 0.5	0.181	11–25
FFA (μEq/L)	97.9 ± 6.9[a]	190.1 ± 25.1[b]	73.7 ± 4.8[a]	0.000	155–350
T-Cho (mg/dL)	97.2 ± 4.6	120.8 ± 9.8	105.6 ± 15.1	0.090	100–180
BUN (mg/dL)	8.8 ± 0.5[a]	11.7 ± 1.1[b]	7.9 ± 1.9[a,b]	0.027	6–27
Glu (mg/dL)	63.7 ± 1.4[a]	58.1 ± 4.4[a]	42.7 ± 5.8[b]	0.002	45–75
Ca (mg/dL)	9.5 ± 9.5	9.2 ± 0.1	9.2 ± 0.1	0.056	9.7–12.4
IP (mg/dL)	6.7 ± 0.3	6.6 ± 0.1	6.2 ± 0.2	0.650	5.6–6.5
Mg (mg/dL)	2.1 ± 0.0[a,b]	2.1 ± 0.1[a]	2.5 ± 0.1[b]	0.022	1.8–2.3
TG (mg/dL)	23.5 ± 2.0[a]	19.1 ± 2.4[a,b]	12.9 ± 1.1[b]	0.039	0–14
Vit. A (IU/dL)	134.1 ± 4.9	138.8 ± 7.1	137.7 ± 9.3	0.838	>80

Vit. E (IU/dL)	190.1 ± 20.8[a]	396.3 ± 37.9[b]	318.7 ± 40.7[b]	0.000	>105
3HB (μmol/L)	419.0 ± 22.2[a]	508.5 ± 59.4[a]	1115.9 ± 268.6[b]	0.000	0–1000
TP (g/dL)	6.8 ± 0.1[a]	6.7 ± 0.1[a,b]	6.3 ± 0.1[b]	0.034	5.7–8.1
Alb (g/dL)	3.5 ± 0.1	3.3 ± 0.1	3.6 ± 0.0	0.171	2.1–3.6
AG ratio	1.1 ± 0.4[a]	1.0 ± 0.1[a]	1.3 ± 0.4[b]	0.013	0.7–1.2

ZEN: zearalenone; AMH: anti-Müllerian hormone; SAA: serum amyloid A; GOT: glutamate-oxaloacetate transaminase; GGT: gamma-glutamyl transpeptidase; FFA: free fatty acid; T-Cho: total cholesterol; BUN: blood urea nitrogen; Glu: glucose; Ca: calcium; IP: inorganic phosphorus; Mg: magnesium; TG: triglycerides; Vit. A: vitamin A; Vit. E: vitamin E; 3HB: 3-hydroxybutyrate; TP: total protein; Alb: albumin; AG: albumin/globulin ratio. *Reference ranges of biochemical analysis reported by Watanabe et al. (2013).

Table S2. Results of urinary ZEN concentration and serum biochemical analysis for herd 3 as a preliminary monitoring.

Parameters	Herd 3-1st (*n*=3)*	Herd 3-2nd (*n*=7)**	*P*-value	Reference value***
ZEN (pg/mg/Cre)	4575.3 ± 927.2 (*n*=2)	4733.8 ± 42.3 (*n*=2)		
AMH (pg/mL)	627.0 ± 110.8	1603.0 ± 279.8	0.061	15–2000
SAA (mg/L)	1.2 ± 0.3	7.5 ± 4.9	0.435	<6.5
GOT (IU/L)	57.8 ± 2.7	62.7 ± 4.8	0.542	35–80
GGT (IU/L)	15.5 ± 0.5	23.5 ± 3.1	0.141	11–25
FFA (μEq/L)	73.7 ± 4.8	436.1 ± 146.0	0.003	155–350
T-Cho (mg/dL)	105.6 ± 15.1	104.4 ± 9.4	0.945	100–180
BUN (mg/dL)	7.9 ± 1.9	14.5 ± 0.6	0.003	6–27
Glu (mg/dL)	42.7 ± 5.8	67.0 ± 4.0	0.010	45–75
Ca (mg/dL)	9.2 ± 0.1	8.4 ± 0.3	0.113	9.7–12.4
IP (mg/dL)	6.2 ± 0.2	8.2 ± 0.5	0.032	5.6–6.5
Mg (mg/dL)	2.5 ± 0.1	2.1 ± 0.1	0.063	1.8–2.3
TG (mg/dL)	12.9 ± 1.1	13.3 ± 1.8	0.880	0–14
Vit. A (IU/dL)	137.7 ± 9.3	128.3 ± 5.5	0.391	>80
Vit. E (IU/dL)	318.7 ± 40.7	355.0 ± 40.4	0.610	>105
3HB (μmol/L)	1115.9 ± 268.6	286.8 ± 27.6	0.001	0–1000
TP (g/dL)	6.3 ± 0.1	7.5 ± 0.2	0.003	5.7–8.1
Alb (g/dL)	3.6 ± 0.0	3.8 ± 0.1	0.395	2.1–3.6
AG ratio	1.3 ± 0.4	1.1 ± 0.1	0.081	0.7–1.2

* Conducted in December 2021. ** conducted at May 2022, all analyzed by T-test.

ZEN: zearalenone; AMH: anti-Müllerian hormone; SAA: serum amyloid A; GOT: glutamate-oxaloacetate transaminase; GGT: gamma-glutamyl transpeptidase; FFA: free fatty acid; T-Cho: total cholesterol; BUN: blood urea nitrogen; Glu: glucose; Ca: calcium; IP: inorganic phosphorus; Mg: magnesium; TG: triglycerides; Vit. A: vitamin A; Vit. E: vitamin E; 3HB: 3-hydroxybutyrate; TP: total protein; Alb: albumin; AG: albumin/globulin ratio. ***Reference ranges of biochemical analysis reported by Watanabe et al. (2013).

Table S3. The time-series trends of all the biochemical test results (linear mixed-model analysis).

Parameter	Day	LS mean	95% CI		*P*-value
AMH	⊿ Day 20 (from Day 0)	0.75	0.71	0.80	<0.001
	⊿ Day 40 (from Day 0)	0.80	0.75	0.84	<0.001
	⊿ Day 40 (from Day 20)	1.06	1.00	1.13	0.178
SAA	⊿ Day 20 (from Day 0)	1.31	0.67	2.53	>0.999
	⊿ Day 40 (from Day 0)	0.95	0.49	1.85	>0.999
	⊿ Day 40 (from Day 20)	0.73	0.38	1.41	>0.999
GOT	⊿ Day 20 (from Day 0)	1.21	1.11	1.32	<0.001
	⊿ Day 40 (from Day 0)	0.95	0.87	1.04	0.753
	⊿ Day 40 (from Day 20)	0.79	0.72	0.86	<0.001
GGT	⊿ Day 20 (from Day 0)	1.04	0.97	1.12	0.672
	⊿ Day 40 (from Day 0)	1.09	1.02	1.17	0.042
	⊿ Day 40 (from Day 20)	1.05	0.98	1.12	0.584
FFA	⊿ Day 20 (from Day 0)	0.16	0.11	0.23	<0.001
	⊿ Day 40 (from Day 0)	1.18	0.81	1.71	>0.999
	⊿ Day 40 (from Day 20)	7.57	5.19	11.03	<0.001
T-Cho	⊿ Day 20 (from Day 0)	0.83	0.78	0.89	<0.001
	⊿ Day 40 (from Day 0)	1.13	1.06	1.21	<0.001
	⊿ Day 40 (from Day 20)	1.36	1.28	1.45	<0.001
BUN	⊿ Day 20 (from Day 0)	0.80	0.67	0.97	0.077
	⊿ Day 40 (from Day 0)	1.81	1.49	2.18	<0.001
	⊿ Day 40 (from Day 20)	2.25	1.86	2.71	<0.001
Glu	⊿ Day 20 (from Day 0)	0.75	0.68	0.83	<0.001
	⊿ Day 40 (from Day 0)	1.01	0.92	1.11	>0.999
	⊿ Day 40 (from Day 20)	1.34	1.22	1.47	<0.001

Ca	⊿ Day 20 (from Day 0)	1.05	1.02	1.09	0.004
	⊿ Day 40 (from Day 0)	1.02	0.99	1.05	0.854
	⊿ Day 40 (from Day 20)	0.96	0.94	1.00	0.079
IP	⊿ Day 20 (from Day 0)	0.81	0.75	0.88	<0.001
	⊿ Day 40 (from Day 0)	1.11	1.02	1.21	0.042
	⊿ Day 40 (from Day 20)	1.37	1.26	1.49	<0.001
Mg	⊿ Day 20 (from Day 0)	1.10	1.03	1.19	0.029
	⊿ Day 40 (from Day 0)	1.10	1.02	1.18	0.037
	⊿ Day 40 (from Day 20)	1.00	0.93	1.07	>0.999
TG	⊿ Day 20 (from Day 0)	0.91	0.68	1.22	>0.999
	⊿ Day 40 (from Day 0)	0.99	0.74	1.33	>0.999
	⊿ Day 40 (from Day 20)	1.09	0.82	1.46	>0.999
Vit. A	⊿ Day 20 (from Day 0)	0.79	0.73	0.84	<0.001
	⊿ Day 40 (from Day 0)	0.71	0.66	0.75	<0.001
	⊿ Day 40 (from Day 20)	0.90	0.84	0.96	0.007
Vit. E	⊿ Day 20 (from Day 0)	0.63	0.57	0.70	<0.001
	⊿ Day 40 (from Day 0)	0.96	0.87	1.07	>0.999
	⊿ Day 40 (from Day 20)	1.52	1.37	1.69	<0.001
3HB	⊿ Day 20 (from Day 0)	1.84	1.39	2.43	<0.001
	⊿ Day 40 (from Day 0)	1.44	1.09	1.90	0.034
	⊿ Day 40 (from Day 20)	0.78	0.59	1.03	0.249
TP	⊿ Day 20 (from Day 0)	0.95	0.93	0.97	<0.001
	⊿ Day 40 (from Day 0)	1.01	0.99	1.03	0.661
	⊿ Day 40 (from Day 20)	1.06	1.04	1.08	<0.001
Alb	⊿ Day 20 (from Day 0)	0.99	0.96	1.01	0.826
	⊿ Day 40 (from Day 0)	1.06	1.03	1.09	<0.001
	⊿ Day 40 (from Day 20)	1.06	1.03	1.09	<0.001

A/G	⊿ Day 20 (from Day 0)	1.07	1.03	1.12	0.007
	⊿ Day 40 (from Day 0)	1.09	1.04	1.14	<0.001
	⊿ Day 40 (from Day 20)	1.02	0.98	1.06	>0.999

AMH: anti-Müllerian hormone; SAA: serum amyloid A; GOT: glutamate-oxaloacetate transaminase; GGT: gamma-glutamyl transpeptidase; FFA: free fatty acid; T-Cho: total cholesterol; BUN: blood urea nitrogen; Glu: glucose; Ca: calcium; IP: inorganic phosphorus; Mg: magnesium; TG: triglycerides; Vit. A: vitamin A; Vit. E: vitamin E; 3HB: 3-hydroxybutyrate; TP: total protein; Alb: albumin; AG: albumin/globulin ratio.

Table S4. The time-series trends of all SCFA test results (linear mixed model analysis).

Parameter	Day	LS mean	95% CI		*P*-value
Formic acid	⊿ Day 20 (from Day 0)	1.30	1.05	1.62	0.059
	⊿ Day 40 (from Day 0)	1.56	1.25	1.93	<0.001
	⊿ Day 40 (from Day 20)	1.20	0.97	1.48	0.285
Acetic acid	⊿ Day 20 (from Day 0)	0.91	0.77	1.08	0.804
	⊿ Day 40 (from Day 0)	0.90	0.76	1.06	0.610
	⊿ Day 40 (from Day 20)	0.99	0.83	1.17	>0.999
Propionic acid	⊿ Day 20 (from Day 0)	0.61	0.49	0.77	<0.001
	⊿ Day 40 (from Day 0)	0.55	0.44	0.69	<0.001
	⊿ Day 40 (from Day 20)	0.90	0.71	1.13	>0.999
Isobutyric acid	⊿ Day 20 (from Day 0)	0.54	0.39	0.75	0.001
	⊿ Day 40 (from Day 0)	0.40	0.29	0.56	<0.001
	⊿ Day 40 (from Day 20)	0.74	0.53	1.02	0.188
Butyric acid	⊿ Day 20 (from Day 0)	0.76	0.64	0.91	0.011
	⊿ Day 40 (from Day 0)	1.13	0.94	1.35	0.532
	⊿ Day 40 (from Day 20)	1.48	1.24	1.77	<0.001
2-Methylvaleric acid	⊿ Day 20 (from Day 0)	1.02	0.78	1.33	>0.999
	⊿ Day 40 (from Day 0)	0.53	0.40	0.69	<0.001
	⊿ Day 40 (from Day 20)	0.51	0.39	0.67	<0.001
Isovaleric acid	⊿ Day 20 (from Day 0)	0.86	0.63	1.17	0.977
	⊿ Day 40 (from Day 0)	0.71	0.53	0.97	0.095
	⊿ Day 40 (from Day 20)	0.83	0.61	1.13	0.683
Valeric acid	⊿ Day 20 (from Day 0)	1.01	0.76	1.34	>0.999
	⊿ Day 40 (from Day 0)	0.76	0.57	1.00	0.151
	⊿ Day 40 (from Day 20)	0.75	0.57	0.99	0.139

Isocaproic acid	⊿ Day 20 (from Day 0)	0.67	0.48	0.94	0.064
	⊿ Day 40 (from Day 0)	0.51	0.36	0.72	<0.001
	⊿ Day 40 (from Day 20)	0.77	0.55	1.08	0.371
Hexanoic acid (caproic acid)	⊿ Day 20 (from Day 0)	1.09	0.93	1.27	0.821
	⊿ Day 40 (from Day 0)	0.87	0.75	1.02	0.260
	⊿ Day 40 (from Day 20)	0.80	0.69	0.94	0.018
2-Methylhexanoic acid	⊿ Day 20 (from Day 0)	0.80	0.65	0.99	0.120
	⊿ Day 40 (from Day 0)	0.62	0.51	0.77	<0.001
	⊿ Day 40 (from Day 20)	0.78	0.63	0.95	0.051
Heptanoic acid	⊿ Day 20 (from Day 0)	0.36	0.22	0.60	<0.001
	⊿ Day 40 (from Day 0)	0.16	0.10	0.26	<0.001
	⊿ Day 40 (from Day 20)	0.44	0.26	0.72	0.005

Table S5. Extracted ion chromatogram for individual short fatty acids in GC/MS measurements.

**Fatty acids**	**Extracted ion chromatogram (m/z)***
Acetic acid	56
Propionic acid	57
Isobutyric acid	71
Butyric acid	71
Valeric acid	85
Isocaproic acid	99
Hexanoic acid	99
2-Methylhexanoic acid	113
4-Methylhexanoic acid	113
Heptanoic acid	113
3-Methylpentanoic acid	99

*Extracted ion chromatograms were referred from Priyo et al. [27]

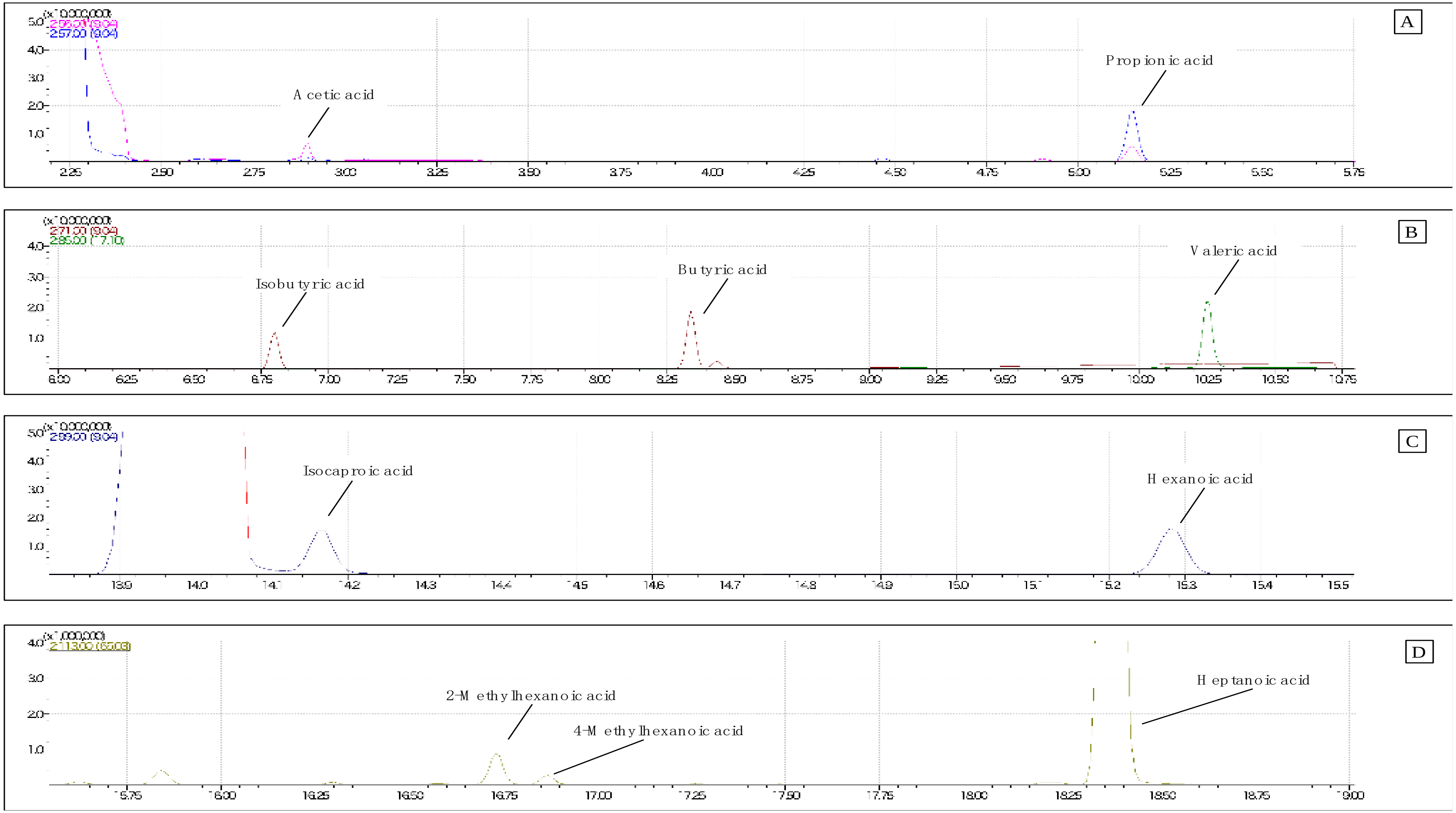

Figure S1. Chromatograms of individual fatty acids in standard solution. A: m/z=56 and 57, B: m/z=71 and 85, C: m/z=99, and D: m/z=113. The horizontal axis shows retention time.
